# Inspiratory Profiles Through Easyhaler Dry Powder Inhaler During Acute Bronchoconstriction

**DOI:** 10.1089/jamp.2024.0045

**Published:** 2025-04-01

**Authors:** Ville A. Vartiainen, Antti Tikkakoski, L. Pekka Malmberg, Liisa Vuotari, Satu Lähelmä, Ulla Sairanen, Mikko Vahteristo, Jussi Karjalainen, Lauri Lehtimäki

**Affiliations:** ^1^ Heart and Lung Center, Helsinki University Hospital, Finland and Faculty of Medicine, University of Helsinki, Helsinki, Finland.; ^2^ Clinical physiology and nuclear medicine, Tampere University Hospital, Faculty of Medicine and Health Technology, Tampere University, Tampere, Finland.; ^3^ Skin and Allergy Hospital, University of Helsinki and Helsinki University Hospital, Helsinki, Finland.; ^4^ Orion Corporation, Espoo, Finland.; ^5^ Allergy Centre, Tampere University Hospital; Faculty of Medicine and Health Technology, Tampere University, Tampere, Finland.

**Keywords:** dry powder inhaler, obstruction, peak inspiratory flow rate

## Abstract

**Background:**

Dry powder inhalers (DPIs) are passive devices, which rely on a patient’s inspiratory effort for drug dispersion and delivery. The aim of this study was to assess how acute bronchoconstriction affects the ability to use Easyhaler DPI in adults.

**Methods:**

This study was conducted as part of a parallel-group clinical trial assessing use of Salbutamol Easyhaler, Budesonide-formoterol Easyhaler and salbutamol pMDI with spacer during a methacholine challenge (MC) test. The inhalations through both Easyhaler variants, the inhaler for the single active substance product (EH-mono) and the inhaler for the combination product (EH-combi), were recorded at baseline and during bronchoconstriction. Peak inspiratory flow (PIF), flow rate acceleration and inhalation volume after PIF were compared to the criteria for successful inhalation.

**Results:**

The study population consisted of 120 adult subjects indicated for MC as a diagnostic test for asthma, with 60 subjects in both Easyhaler arms. With EH-combi 98.3% and 91.4% passed the criteria (PIF ≥30 L/min, inhalation acceleration ≥0.7 L/s^2^, and inhalation volume ≥500 mL after PIF) for successful inhalation at baseline and during bronchoconstriction, respectively. With EH-mono, success rates were 95.0% and 88.1% at baseline and during bronchoconstriction, respectively. The most common reason for not passing the criteria was slow inhalation acceleration. Aside from two subjects using EH-mono during bronchoconstriction, all subjects were able to generate PIF ≥ 30 L/min.

**Conclusions:**

During an acute obstructive event, the vast majority of patients have no difficulty in achieving sufficient PIF, inhalation acceleration, and volume after PIF when using an Easyhaler DPI.

## Introduction

Correct use of inhaler devices is crucial for successful inhalation therapy, but there are distinct differences in the use of different types of inhalers.^1^ Dry powder inhalers (DPIs) are passive devices, which rely on a patient’s inspiratory effort for drug dispersion and delivery, while propellant-containing pressurized metered dose inhalers (pMDIs) use pressurized gas for dispersion but require coordination between inhaler actuation and inhalation.

The required inspiratory flow rate for DPIs to function properly varies between the devices. While optimal flow rate is difficult to define and depends on the specified endpoint (see, for example, Pohlman et al.^2^ and Haidl et al.^3^), many manufacturers have recommendations on minimum peak inspiratory flow rate (PIF) for successful inhalation with their respective devices. In clinical practice, following the inhalation instructions without measuring PIF is regarded as sufficient to ensure correct inhalation technique. As all DPIs utilize the energy associated with inspiratory flow to deagglomerate the drug formulation, there is always at least some flow dependency involved, and higher flow rates yield better deagglomeration. However, higher inspiratory flow rates also increase impaction of drug particles to oropharynx. The optimal inspiratory flow is therefore a balance between sufficient deagglomeration and lower oropharyngeal losses.^4^

In addition to the minimum PIF, inhalation flow rate acceleration, and inhaled volume after PIF have also been proposed as criteria for successful inhalation by Kamin et al.^5,6^ This work was supplemented areview by Haidl et al., where the authors extended the criteria to various other devices based on *in vitro* evidence available.^3^ Most of the DPIs on the market emit the dose during the initial part of the inhalation.^1^ Therefore, it is important that the PIF occurs as early as possible to ensure that sufficient energy is available for the deagglomeration. Sufficient volume after PIF is required to ensure that the deagglomerated drug reaches the distal parts of the airways.

In general, patients seem to have no difficulty in achieving sufficient flow rates.^7^ Haughney et al. studied a population of over 900 asthmatic patients using the In-Check Dial with a flow rate requirement of 30–90 L/min for successful inhalation for DPI resistances with the instruction to breathe in strongly and deeply.^8^ With the highest resistance level, 94% of the patients were able to perform successful inhalation. The success rate with pMDI resistance and flow (20–60 L/min for successful inhalation) was lower (71%) due to the patients inhaling too forcefully. The measurement of inhalation acceleration and volume after PIF needs more sophisticated equipment and are therefore less well studied. Broeders et al. studied 15 patients hospitalized for asthma or chronic obstructive pulmonary disease (COPD) exacerbation.^9^ All of these patients were able to produce an inspiratory flow rate of 30 L/min with medium-low and medium-high resistance DPIs, while inhalation was deemed successful for 14% of the patients with pMDI and 87% for patients with pMDI + spacer. Several DPIs have been compared against pMDI, mainly in adult patients with asthma or COPD, using salbutamol, formoterol, and tiotropium as bronchodilator. No significant difference was found between DPI and pMDI and/or nebulizer when recovery of FEV_1_ was considered as the clinically relevant outcome measure.^10–21^

Inspiratory profiles via Easyhaler have been studied both in healthy volunteers and stable patients with asthma or COPD. Results have been in line with the results obtained with an In-Check Dial. A recent study by Malmberg et al. pooled inspiratory profiles of 102 children with asthma, 185 adults with asthma, and 96 patients with COPD through a medium-high resistance variant of Easyhaler used in the combination products (EH-combi). In all groups, only one subject failed to achieve the sufficient peak inspiratory flow rate of 30 L/min.^22^ Similarly, in the crossover study by Jõgi et al. 100 healthy volunteers and 100 COPD patients were examined with a medium-high resistance EH-combi and a high resistance Easyhaler used in single active substance products (EH-mono). All subjects achieved the sufficient inspiratory flow rate of 30 L/min with both Easyhaler variants.^23^

The aim of this study was to assess how acute bronchoconstriction affects the use of Easyhaler based on PIF, flow rate acceleration, and inhalation volume after PIF in the adult population.

## Materials and Methods

This study was conducted as part of a clinical trial assessing the use of Salbutamol Easyhaler, Budesonide-formoterol Easyhaler, and salbutamol pMDI with spacer during a methacholine challenge (MC) test.^24^ The study was approved by the Tampere University Hospital Ethics Committee (Committee reference: R21100M; EudraCT number: 2021-001573-22; Clinicaltrials.gov: NCT05084222) and conducted in accordance with the Declaration of Helsinki and Good Clinical Practice.

### Participants

The participants of the study were adults (18 years or older) indicated for MC as a diagnostic test for asthma. Eligible subjects demonstrated a FEV_1_ drop of at least 20% during the MC test compared with baseline spirometry. Exclusion included the clinical exclusion criteria of the MC test, the use of forbidden treatments such as any bronchodilating substances before the study visit, or known hypersensitivity to the active substances, or excipients of the study treatments. All participants provided written informed consent to take part in the study.

### Study design

**FIG. 1.**
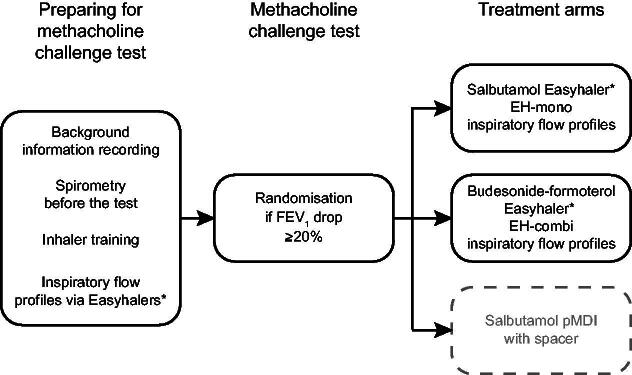
Study design chart for the inhalation study. *Only data from Easyhaler treatment arms were included in this study.

After an FEV_1_ drop of at least 20% in the MC test, eligible participants were randomly allocated by the permutated block method (1:1:1) to receive one of the study treatments, Salbutamol Easyhaler (EH-mono), Ventoline Evohaler with Volumatic spacer, or Budesonide-formoterol Easyhaler (EH-combi). The MC test was conducted according to the current technical standard.^25^ Spirometry was performed, and FEV_1_ was recorded before the MC test, after the inhalation of a saline solution, approximately 2 minutes after each methacholine dose, and 10 minutes after the study treatment administration (Fig. 1). Cumulative doses of methacholine were given in approximately 5-minute intervals until the largest dose, or a drop of at least 20% in FEV_1_, was achieved. Spirometry was conducted in accordance with the American Thoracic Society/European Respiratory Society guideline.^26^

In this work, we are reporting the inspiratory profiles of the study participants who were randomized to either of the Easyhaler arms. The Easyhaler is a reservoir-type device and comes in two varieties, EH-combi (airflow resistance 0.036 $\sqrt{k\mathrm{Pa}}\;\min/L)$ is used in the combination products (budesonide-formoterol and salmeterol-fluticasone propionate), and EH-mono (airflow resistance 0.044 $\sqrt{k\mathrm{Pa}}\;\min/L)$ is used in the products with a single active substance (salbutamol, formoterol, beclomethasone, and budesonide).

Inspiratory profiles were measured through Easyhaler before the MC test with empty inhalers (at baseline) and after an FEV_1_ drop of at least 20% while administering the study drug as a reliever (during bronchoconstriction). The methodology for the measurement of the inspiratory flow profiles has been described in detail elsewhere, and two inspiratory flow curves were recorded for each subject.^27^ Briefly, the inhaler devices were connected with a pneumotachograph (Spiromaster MX, Medikro Ltd, Kuopio, Finland), which allows the measurement of inspiratory parameters while inhaling through the device.

### Statistical methods

PIF, inhalation acceleration, time to PIF (t_max_), inspiratory volume (V) and V after PIF (V_PIF_) were analyzed from the inhalation with higher PIF of the two inhalations at each time point. The inhalation was then compared with the criteria described by Kamin et al. and Haidl et al. (Kamin–Haidl criteria: = PIF ≥30 L/min for Easyhaler, inhalation acceleration ≥0.7 L/s^2^, and inhalation volume ≥500 mL after PIF) for successful inhalation.^3,5,6^ Statistical tests between baseline and bronchoconstriction were performed by paired *t*-test for observed cases.

## Results

A total of 120 participants were randomized to this study, 60 participants in both the EH-mono and EH-combi arms. Due to equipment malfunction, the data required for Kamin–Haidl criteria was not available for two participants during the bronchoconstriction in the EH-combi arm and one participant during the bronchoconstriction in the EH-mono arm. Demographic characteristics of the study population are presented in Table 1.

**Table 1.** Demographic Characteristics and Lung Function of the Study Population

	EH-mono *n* = 60	EH-combi *n* = 60	Total *n* = 120
Sex			
Female	45 (75.0)	36 (60.0)	81 (67.5)
Male	15 (25.0)	24 (40.0)	39 (32.5)
Age	47.1 (16.2)	43.7 (16.9)	45.4 (16.6)
Weight	78.2 (18.8)	82.5 (20.8)	80.3 (19.9)
BMI	27.7 (6.3)	28.5 (6.2)	28.1 (6.3)
Smoking			
Current smoker	9 (15.0)	6 (10.0)	15 (12.5)
Ex-smoker	8 (13.3)	13 (21.7)	21 (17.5)
Never smoked	43 (71.7)	41 (68.3)	84 (70.0)
FEV_1_			
At baseline	2.85 (0.77)	3.09 (0.89)	2.97 (0.84)
During bronchoconstriction	2.09 (0.60)	2.27 (0.66)	2.18 (0.63)
Fev_1_ % of predicted			
At baseline	88.2 (12.0)	89.3 (15.2)	88.7 (13.6)
During bronchoconstriction	64.6 (9.9)	65.9 (12.3)	65.2 (11.2)

Sex, smoking, and race are expressed as *n* (%) and others as mean (SD).

EH-mono, Easyhaler used in products with a single pharmaceutical ingredient; EH-combi, Easyhaler used in combination products.

**FIG. 2.**
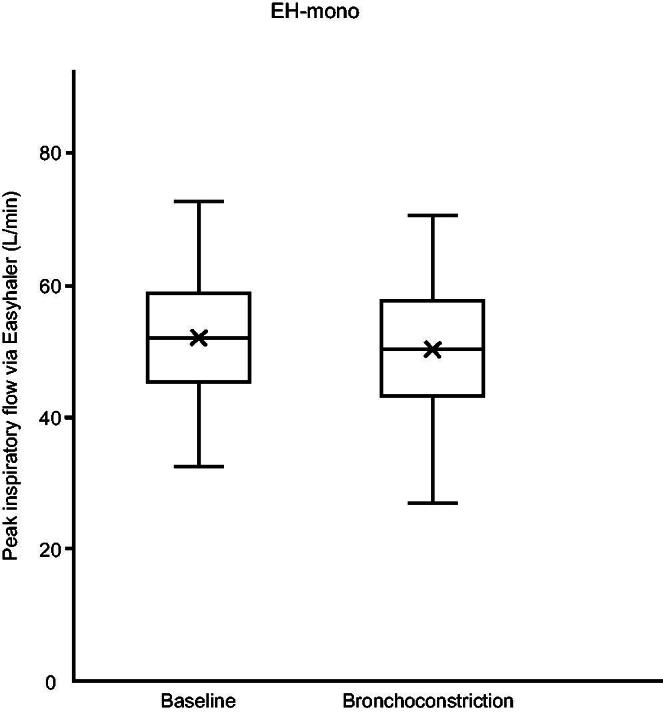
Boxplot of peak inspiratory flow rate (PIF) at baseline and during bronchoconstriction with Easyhaler mono (EH-mono). EH-mono, Easyhaler used in products with a single pharmaceutical ingredient.

**FIG. 3.**
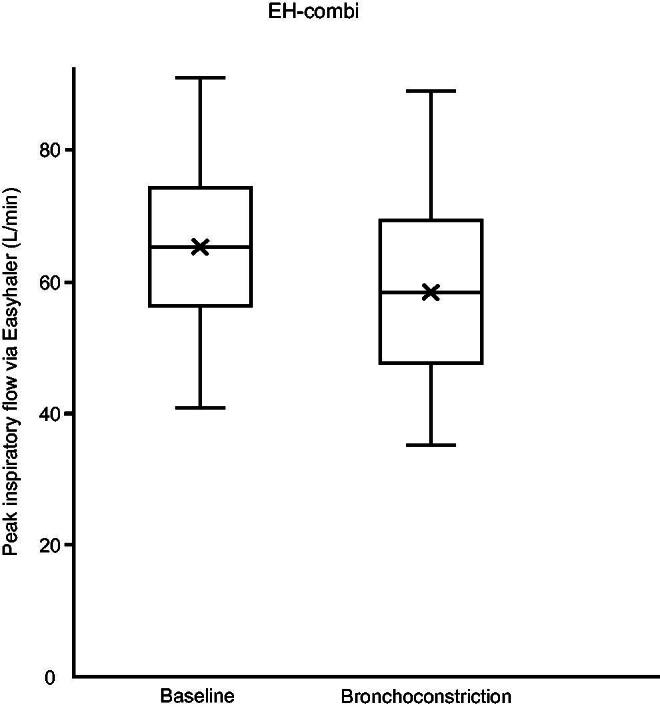
Boxplot of peak inspiratory flow rate (PIF) at baseline and during bronchoconstriction with Easyhaler combi (EH-combi). EH-combi, Easyhaler used in combination products.

Figures 2 and 3 show PIF, and Table 2 shows PIF, V, V_PIF_, inhalation acceleration, and t_max_ through both Easyhaler device varieties at baseline and during obstruction. Aside from two subjects using EH-mono during bronchoconstriction, all subjects were able to generate PIF ≥ 30 L/min. There was a statistically significant difference between PIF and acceleration at baseline and during bronchoconstriction in both Easyhaler variants. The differences between baseline and during bronchoconstriction were insignificant for V_PIF_ and t_max_.

**Table 2.** Peak Inspiratory Flow Rate (PIF), Inspiratory Volume (V), Inspiratory Volume after PIF (V_PIF_), Inhalation Acceleration and Time to PIF from Start of Inhalation (T_max_) at Baseline and during Bronchoconstriction (Mean [Range])

Device variant	Timepoint	PIF (L/min)	V (L)	V_PIF _(L)	Acceleration (L/s^2^)	T_max_ (s)
EH-mono	Baseline (*n* = 60)	52.6 (32.5–72.7)	2.04 (0.73–5.66)	1.59 (0.37–3.96)	2.91 (0.61–7.71)	0.71 (0.25–1.99)
	Bronchoconstriction (*n* = 59)	50.5 (27.1–70.9)**	2.00 (0.75–4.51) NS	1.55 (0.47–3.91) NS	2.57 (0.45–7.81)*	0.75 (0.27–2.03) NS
EH-combi	Baseline (*n* = 60)	65.9 (40.8–91.3)	2.34 (0.65–4.33)	1.80 (0.49–3.89)	3.90 (0.89–8.51)	0.69 (0.29–1.85)
	Bronchoconstriction (*n* = 59)	59.9 (35.0–89.3)**	2.21 (0.43–3.85) NS	1.70 (0.21–3.31) NS	3.44 (0.31–8.08)*	0.76 (0.26–3.23) NS

Statistical significance of difference from baseline denoted by **p < 0.01, *p < 0.05, and NS *p* > 0.05.

The inhalations were assessed according to Kamin–Haidl criteria for success, and the results are presented in Table 3. With EH-combi, 98.3% and 91.4% passed the Kamin–Haidl criteria at baseline and during bronchoconstriction, respectively. With EH-mono, success rates were 95.0% and 88.1% at baseline and during bronchoconstriction, respectively. The most common reason for not passing the Kamin–Haidl criteria was low inhalation acceleration (Table 3). Altogether two participants at baseline for EH-mono and four participants for both device variants in bronchoconstriction did not achieve acceleration ≥0.7 L/s^2^.

**Table 3.** Success according to Kamin–Haidl Criteria. Permutations without Occurrences Are Omitted

	Acceleration (≥0.7 L/s^2^)	PIF (≥30 L/min)	V_PIF_ (≥0.5 L)	Kamin-Haidl criteria	Frequency
EH-mono					
	Baseline *n* = 60	Yes	Yes	Yes	Yes	57
	* *	Yes	Yes	No	No	1
		No	Yes	Yes	No	2
	Bronchoconstriction *n* = 59	Yes	Yes	Yes	Yes	52
	* *	Yes	Yes	No	No	1
		No	Yes	Yes	No	4
		Yes	No	Yes	No	2
EH-combi						
	Baseline *n* = 60	Yes	Yes	Yes	Yes	59
	* *	Yes	Yes	No	No	1
	Bronchoconstriction *n* = 58	Yes	Yes	Yes	Yes	53
		Yes	Yes	No	No	1
		No	Yes	Yes	No	4

Acceleration, inhalation acceleration; PIF, peak inspiratory flow rate; V_PIF_, inspiratory volume after PIF.

## Discussion

In general, the differences in measured parameters between baseline and bronchoconstriction were small, and the patients performed well with both device variants. While the differences in PIF between baseline and obstructive inhalations were statistically significant, they were small and therefore not clinically relevant. PIF independent dosing with Easyhaler has been shown with a clinically relevant PIF rate range.^28–30^ The observed flow rate dependency of the dose with both Easyhaler variants was small compared to the general *in vitro* equivalence requirement of ±15% in the European medicines agency (EMA) guideline for clinical documentation for orally inhaled products.^31^ The changes in PIF seen in this study were negligible compared with the range for which PIF independent dosing has been shown previously. Only two subjects (1.67%) were unable to generate the sufficient flow rate with EH-mono after the MC test, and similar results have been reported with another DPI in the same device resistance range.^32^

The vast majority of participants had sufficient inhalation acceleration for a successful inhalation maneuver. However, in this study, inhalation acceleration was the most common reason for a suboptimal inspiratory maneuver according to Kamin–Haidl criteria.^5,6^ At the baseline, the observed accelerations were approximately 10%–20% lower than previously reported for patients with asthma.^33^ In reservoir and blister-type devices, most of the dose emission occurs during the first and second inhalation and therefore, sufficient acceleration is needed for the efficient deagglomeration of the drug formulation. Capsule-based devices release the drug more slowly over a longer time, and they are not expected to be as sensitive to changes in acceleration.^1^

In this study, there were only a few failures due to inhaled volume after PIF, and the observed difference between the baseline and during bronchoconstriction was not significant. The total inhalation volume through both devices was comparable to what has been reported previously.^33^ During the drug delivery, sufficient volume is required to carry the drug from the device to the site of action after deagglomeration. With reservoir and blister-type devices, the drug release is fast, and they are expected to be less sensitive to volume after PIF than capsule-type devices, where the drug release is slower.^1^

The results at baseline are in line with those reported previously by Haikarainen et al., where among 287 patients with asthma and 96 patients with COPD, requirements for successful inhalation according to the Kamin–Haidl criteria were met with EH-combi in 96.1% and with EH-mono in 92.6% of patients.^33^ While we did observe differences in some inspiratory parameters between baseline and bronchoconstriction, almost all patients satisfied the Kamin–Haidl criteria for correct inhalation despite the clinical condition, and the failures observed are not likely to be critical in the sense that they would prevent the patient from getting any drug. Indeed, while the *in vitro* results suggest that the Easyhaler demonstrates some flow-dependent dose emission, it was effective also at low inhalation flows and inhaled volumes.^34^

The success rates for inhalations are in line with previously reported results from Haughney et al., even though more parameters were recorded and actual inhaler devices were used instead of In-Check Dial in our study.^8^ If only PIF was considered, subjects in our study performed better than those studied by Haughney et al. These results confirm that the Easyhaler inhaler can be used during bronchoconstriction and provides an efficient delivery of medication, as shown earlier, for both adults and children.^24,35,36^ The study population of this work consisted of patients referred to MC, which is a minority of all patients receiving a diagnosis for asthma in Finland. The test is typically done if other pulmonary function tests fail to provide the diagnosis, but asthma is still deemed likely by the clinician. However, the patients did experience verified bronchoconstriction and are a group for which bronchodilators are indicated. The measurements for FEV_1_ followed the standard protocol of methacholine challenge test, and the number of spirometries varies depending on the required number of methacholine doses. While there is a possibility of increasing fatigue caused by repeated forced exhalation maneuvers, it has not been seen as problematic in the clinical interpretation of the methacholine challenge in general. Therefore, we do not expect it to play a significant role in the observed fall in FEV_1_. However, if our setup is affected by fatigue, inhalation profiles in real life without repetitive spirometries would likely be even better than in our study.

## Conclusions

In this work, we have shown that even during an acute obstructive event, the vast majority of patients have no difficulty in achieving sufficient PIF, inhalation acceleration, or V_PIF_ in using an Easyhaler DPI. While we observed a statistically significant decrease in PIF through the inhaler at bronchoconstriction compared to the baseline, it was small and not clinically significant.

## Abbreviations Used

DPI: dry powder inhaler
EH-combi: Easyhaler used in combination products
EH-mono: Easyhaler used in products with a single pharmaceutical ingredient
MC: methacholine challenge
PIF: peak inspiratory flow rate
pMDI: pressurized metered dose inhaler
t_max_: time to PIF from start of inhalation
V: inspiratory volume
V_PIF_: inspiratory volume after PIF
